# Monitoring coagulation-fibrinolysis activation prompted timely diagnosis of hemophagocytic lymphohistiocytosis-related disseminated intravascular coagulation

**DOI:** 10.1186/s12959-021-00338-y

**Published:** 2021-11-04

**Authors:** Liqin Ling, Xunbei Huang, Chaonan Liu, Juan Liao, Jing Zhou

**Affiliations:** Department of Laboratory Medicine, West China Hospital, Sichuan University, No. 37 Guo Xue Alley, Sichuan 610041 Chengdu, China

**Keywords:** Coagulation, Fibrinolysis, Hemophagocytic lymphohistiocytosis, Disseminated intravascular coagulation

## Abstract

**Background:**

Timely diagnosis of disseminated intravascular coagulation (DIC) in hemophagocytic lymphohistiocytosis (HLH) patients is crucial but challenging, as HLH interferes with the results of the laboratory tests included in the DIC score system.

**Case presentation:**

Here, we reported a case of lymphoma-associated HLH, in which coagulation-fibrinolysis activation /inhibition markers (TAT, tPAIC, and PIC), prompted timely diagnosis of early stage DIC (initial phase of microvascular thrombosis, yet non-overt), prior to the development of organ failures and/or bleedings.

**Conclusions:**

This report highlights the importance of the implementation of new biomarkers (such as TAT, tPAIC, and PIC), into the diagnostic work-up for coagulation disorders. These biomarkers are directly suggestive of microthrombus formation, therefore they can be of paramount importance in diagnosing DIC with complicated etiologies, such as hematological diseases-related DIC.

**Supplementary Information:**

The online version contains supplementary material available at 10.1186/s12959-021-00338-y.

## Background

Disseminated intravascular coagulation (DIC) has been observed in hemophagocytic lymphohistiocytosis (HLH) patients in nearly 50 % of cases [1, 2]. DIC is approved to be associated with higher mortality in HLH patients [2]. Therefore, it is vital to diagnose DIC early in HLH patients to take timely treatments options. However, it is challenging to diagnose DIC in HLH patients, as HLH has been shown to interfere with the major indices used in the DIC score system, including platelet count and fibrinogen concentration [1, 2]. Therefore, much effort has been given to the development of more specific biomarkers to support DIC diagnosis. Here, we have discussed the value of coagulation-fibrinolysis activation /inhibition markers in the early diagnosis of DIC before the manifestation of obvious clinical symptoms.

## Case presentation

A 47-year-old man was diagnosed with CD30^+^ lymphoma with HLH complications one year ago. He had already been through four courses of chemotherapy, including one course of CHOP (cyclophosphamide, doxorubicin, vincristine, and prednisone) and three courses of BV-CHP (brentuximab vedotin in combination with cyclophosphamide, doxorubicin, and prednisone). He was scheduled for his fifth chemotherapy on Nov 19, 2020. However, on the day of the hospitalization, his HLH was still significantly active (interleukin-2 receptor 32,560 U /mL, ferritin 12,573 ng /mL). Moreover, he was febrile and thus suspected with infection. Therefore, his clinicians decided to suspend his chemotherapy until his HLH and infection got controlled with corresponding treatments. The patient was therefore given etoposide and dexamethasone (treatment of HLH in accordance to the HLH-2004 protocol [3]), antibiotics (treatment of infection), and further symptomatic treatments.

However, his condition was not improving during this hospitalization. He had started to demonstrate refractory fibrinogenemia since Nov 22, 2020, and he was given fibrinogen infusion accordingly. The cause of his fibrinogenemia was thought to be primary fibrinolysis at that time, considering that primary fibrinolysis frequently occurs in HLH patients due to a massive endogenous release of tissue plasminogen activator from activated monocytes [1]. Therefore, antifibrinolytic therapy was commenced since Nov 27, 2020 (tranexamic acid 1 g per day) to prevent bleeding complications (the patient had suffered gastrointestinal hemorrhage during his last chemotherapy). Unfortunately, his fibrinogenemia was still rapidly progressing, his plasma fibrinogen concentration decreased to an undetectable level (<0.5 g/L) on Dec 13, 2020, even though the antifibrinolytic therapy was effective (his plasma levels of fibrinogen/fibrin degradation products, including FDP and D-dimer, had been decreasing since the antifibrinolytic therapy was commenced ) (shown in supplementary Table 1).

During this period, his platelet count was decreasing over time (from 23 × 10^9^ /L on Nov 22, to 2 × 10^9^ /L on Dec 13), his red blood cell (RBC) count was slightly decreasing (from 2.42 × 10^12^ /L on Nov 22, to 2.07 × 10^12^ /L on Dec 13 ), and his white blood cell (WBC) count was fluctuating due to multiple infusions of recombinant human granulocyte colony-stimulating factor (G-CSF) (shown in supplementary Table 1). And there had been no clinical signs of thrombosis or bleeding, or liver dysfunction yet.

It seems incomprehensible that his fibrinogenemia was not accompanied by either thrombosis /hyperfibrinolysis (excessive consumption) or hepatic impairment (decreased synthesis). His fibrinogenemia could not be improved until we found the answer to this puzzling question.

## Discussion and Conclusions

Acquired fibrinogenemia is most frequently caused by decreased synthesis, hemodilution, or excessive consumption [4]. In our patient, decreased synthesis was excluded, because on Dec 13, 2020, his coagulation factor VII (103 %), a marker of hepatocellular synthetic function [5], was still within the normal range (70-150 %). Besides, his albumin level had been stable since hospitalization (around 30 g/L), although it was lower than the normal range (40-55 g/L), it could be explained by his overall unhealthy status. Hemodilution was also ruled out as there wasn’t any presence of a massive fluid infusion. Therefore, excessive consumption was suspected. Both thrombin and plasmin can use fibrinogen as a substrate, consequently both coagulation activation and fibrinolysis activation can result in excessive consumption of fibrinogen [6, 7]. Since fibrinolysis was inhibited by tranexamic acid in this patient, systemic coagulation activation, which can result in microvascular thrombosis (the early or compensatory stage of DIC) [8], seemed to be a reasonable cause of his fibrinogenemia.

However, diagnosing DIC in this patient was very challenging, especially during the early stage (initial phase of microvascular thrombosis). Clinically, microvascular thrombosis is not readily apparent as clot formation primarily involves the microvasculature. It usually gets noticed during the presentation of organ failures [9], when it is already too late. Therefore, timely diagnosis of DIC, especially at an early stage, should not be relied on clinical manifestations. The diagnosis of DIC was further exacerbated in this case due to the issues that occurred in the standard laboratory diagnostic work-up. Primarily, his fibrinogenemia had already impaired global coagulation parameters (prothrombin time, PT; international normalized ratio, INR; activated partial thromboplastin time, aPTT; and thrombin time, TT), which are the most prevalent tests implemented in a coagulation lab. Secondly, his antifibrinolytic therapy had influenced FDP /D-dimer testing, which could also be indicative of ongoing coagulation activation otherwise [10]. Lastly and most importantly, platelet phagocytosis in his HLH background could confound thrombocytopenia caused by coagulation activation [11], diminishing the value of thrombocytopenia.

Theoretically, regardless of the underlying conditions, unbalanced coagulation-fibrinolysis activation (more thrombin, less plasmin) is inherent to the early stage of DIC [9], which is a prerequisite for microthrombus formation. Thus coagulation-fibrinolysis activation /inhibition markers could be specific measures of DIC. Consistent with this theory, thrombin activation marker (thrombin-antithrombin complex, TAT, normal range: < 4.0 ng /mL) and plasmin inhibition marker (tissue plasminogen activator-plasminogen activator inhibitor complex, t-PAIC, normal range: < 10.5 ng /mL) were elevated in this patient on Dec 13, 2020 (TAT, 52 ng /mL; t-PAIC, 95.6 ng /mL ), while plasmin activation marker ( plasmin-α2-plasmin inhibitor complex, PIC, normal range: < 0.8 µg /mL) was within the normal range (PIC, 0.27 µg /mL). These results highly suggested a prothrombotic state (early stage of DIC) in this patient, even though there was no specific clinical signs yet.

**Fig. 1:**
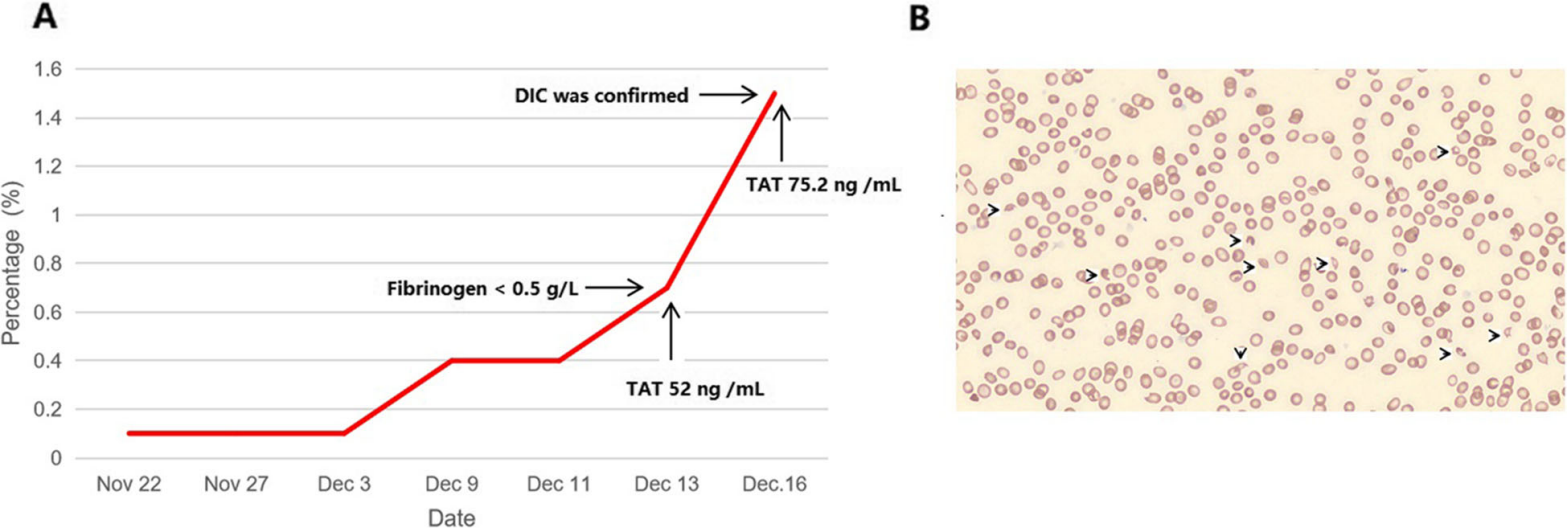
Schistocytes in the peripheral smear as a supplementary evidence of microthrombus formation during early stage DIC. **A** the percentage of schistocyte was increasing over time; **B** a representative image of schistocytes in the peripheral smear on Dec 16, 2020, black arrows (schistocytes)

**Fig. 2:**
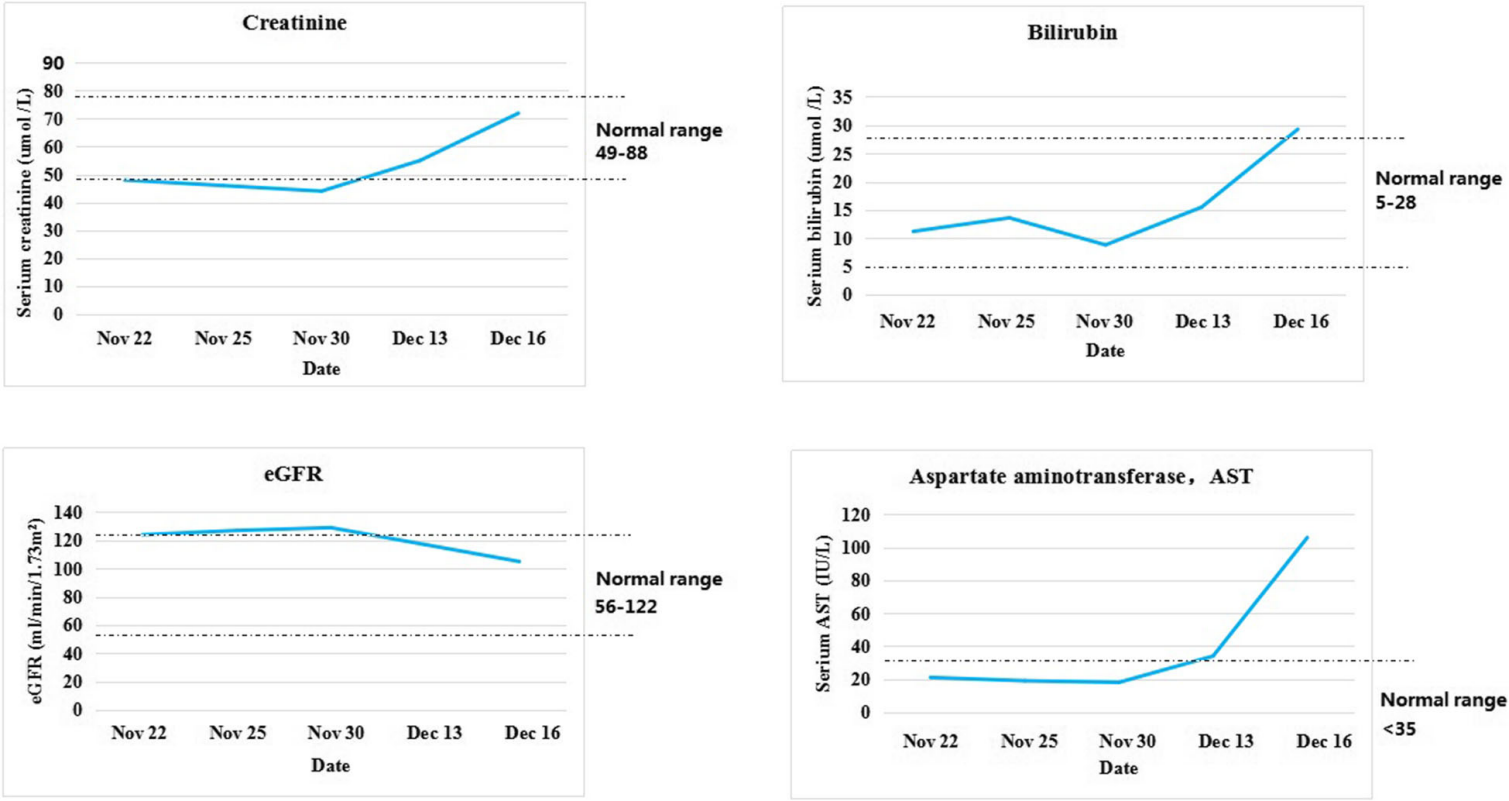
Change of some serum biochemical indicators as another supplementary evidence of microthrombus formation during early stage DIC. All parameters seemed stable until Dec 13, 2020, but got worse on Dec 16, 2020, indicating organ impairments

However, the diagnostic value of these markers in DIC has not been well-defined, DIC diagnosis in this case could not be confirmed yet, besides, the patient refused to take a biopsy. Anyway, these results reminded us to focus on the course of the disease in this patient, which resulted in finding additional evidence to support a diagnosis of early DIC stage in this case. First, we performed tests for his coagulation factors on Dec 16, 2020. We found that several coagulation factors were diminished ( FV 55 %, FIX 69 %, FX 46 %, FXI 46 %, FXII 43 %) and TAT was more elevated (75.2 ng /mL), suggesting that thrombin was chronically consuming coagulation factors in the patient, indicating a chronic systemic coagulation activation. Secondly, after carefully reviewing his routine blood test results, we found an increasing percentage of schistocytes since his hospitalization (from 0.1 % on Nov 22, to 1.5 % on Dec 16, shown in Fig. 1), which was an important marker of microthrombus formation [12]. Lastly, even though there was no prevalent clinical manifestations of renal/liver dysfunction, we found that renal/liver function markers were changing over time which indicated organ impairments (not classified as organ failures as most of the markers were still within the normal ranges) (shown in Fig. 2). Eventually, the diagnosis of DIC in this patient was confirmed on Dec 16, 2020. However, there was still no prevalent clinical signs of renal/liver dysfunction. Our report here demonstrates that coagulation-fibrinolysis activation /inhibition markers could help to prompt timely diagnosis of the early stage of DIC before it could progress to organ failures and/or bleedings.

After his DIC was diagnosed, his antifibrinolytic therapy was stopped immediately, so that his fibrinolysis could get ameliorated, and therefore avoid more microvascular thrombosis. Besides, his DIC could signify that his HLH was not under control with treatments aimed at HLH itself. His clinicians decided to start his fifth chemotherapy course immediately, because his lymphoma was the root cause of his HLH. It should be noted that the fifth chemotherapy was ABVD course (adriamycin, bleomycin, vinblastin, dacarbazine), then changed to AVD because the patient was allergic to bleomycin. This course was different from his previous chemotherapies, because his clinicians thought that prior chemotherapies might not be practical due to his worsening condition. The patients’ condition improved after these treatments, several days after he finished his fifth chemotherapy, on Dec 24, 2020, his TAT decreased to 19.6 ng /mL, PIC increased to 2.49 µg /mL and and t-PAIC decreased to 15.6 ng /mL. And his fibrinogenemia had been ameliorated ever since the above-mentioned managements were instituted (shown in supplementary Table 1). Other laboratory test results were also improving, including platelet counts, renal/liver function markers, et al. (shown in supplementary Table 1).

It is challenging to diagnose DIC early in patients with HLH, as HLH interferes with the major indices used in the DIC score system. HLH itself shares two coagulation abnormalities (thrombocytopenia, hypofibrinogenemia) with DIC [1, 2]. Besides, as mentioned above, HLH related antifibrinolytic therapy can diminish the diagnostic value of FDP/D-dimer. In addition to HLH, several other clinical conditions may also interfere with the current diagnostic work-up for DIC. Examples include hepatic cirrhosis (instead of coagulation activation, thrombocytopenia might be caused by hypersplenism, coagulation factor deficiency (including hypofibrinogenemia) might caused by decreased synthesis) [13], or bone marrow suppression (thrombocytopenia due to less thrombopoiesis) [14], or heparin-induced thrombocytopenia (thrombocytopenia due to excess platelet consumption) [15].

To compensate for the aforementioned issues, parameters directly suggestive of coagulation activation might be more relevant [9, 16], such as prothrombin fragment 1 + 2 (F1 + 2), *ex vivo* thrombin generation assay, and TAT testing. Besides, disturbed fibrinolysis is also an essential contributor to the progress of DIC. In the early phase of DIC, fibrinolysis might be impaired so that microthrombus forms, but fibrinolysis might be over-activated at the late stage of DIC, which leads to bleeding tendency [9, 17]. Therefore, markers of fibrinolysis should also be considered, such as t-PAIC and PIC. Tests for TAT, PIC, t-PAIC have already been standardized, automated, and implemented in some routine coagulation laboratories in Asian countries such as China and Japan [18]. We measured these markers via qualitative chemiluminescence enzyme immunoassay performed on HISCL automated analyzers (HISCL-5000, Sysmex, Japan) [18]. TAT is considered a sensitive marker of thrombin generation, while PIC is an indicator of plasmin generation. There are already studies demonstrating that TAT could predict DIC development in sepsis patients at inclusion [18, 19], TAT was higher in patients who subsequently developed DIC than in patients who did not develop DIC. Moreover, TAT in combination with PIC was more potent than TAT alone [20], compared to patients with elevated TAT plus elevated PIC, which indicated a new balance of thrombin/plasmin generation, patients with elevated TAT alone (PIC was not elevated) had a poorer prognosis (higher risk to develop multiple organ failures). This is consistent with the theory that unbalanced coagulation-fibrinolysis activation is inherent to DIC development [9].

It has been proved that the prognosis of DIC becomes very poor if not treated timely before clinical symptoms manifest (organ failures and/or bleedings) [21, 22]. Currently, there are several well-known scoring systems for DIC diagnosis, all these systems include laboratory indices like platelet count, PT, fibrinogen, FDP/D-dimer [9]. However, as discussed above, these markers can be easily influenced, making it challenging to diagnose DIC at an early stage. Our report here demonstrated that coagulation-fibrinolysis activation /inhibition markers (TAT, t-PAIC, PIC) helped diagnose DIC at an early stage despite some indices (platelet count, PT, FDP/D-dimer) in the DIC score system being influenced. Combined with literature reviewing, it would be prudent to pioneer the implementation of coagulation-fibrinolysis activation /inhibition markers into the diagnostic work-up for DIC so that timely diagnosis and personalized management of DIC can be achieved. In addition, the Japanese Society on thrombosis and hemostasis is proposing a new DIC diagnostic criteria, in which TAT is included as a major index [22].

## Supplementary Information

**Additional file 1: Supplementary Table 1.** Laboratory tests results.

## Data Availability

All data generated or analysed during this study are included in this published article.

## Abbreviations

DIC: Disseminated intravascular coagulation
HLH: Hemophagocytic lymphohistiocytosis
TAT: Thrombin-antithrombin complex
tPAIC: Tissue plasminogen activator-plasminogen activator inhibitor complex
PIC: Plasmin-α2-plasmin inhibitor complex
FDP: Fibrinogen/fibrin degradation products
PT: Prothrombin time
aPTT: Activated partial thromboplastin time
TT: Thrombin time
INR: International normalized ratio
CHOP: Cyclophosphamide, doxorubicin, vincristine, and prednisone
BV-CHP: Brentuximab vedotin in combination with cyclophosphamide, doxorubicin, and prednisone
ABVD: Adriamycin, bleomycin, vinblastin, dacarbazine
