# Supplementary material for: Monitoring coagulation-fibrinolysis activation prompted timely diagnosis of hemophagocytic lymphohistiocytosis-related disseminated intravascular coagulation
Source: Thromb J. 2021 Nov 4;19:82. doi: 10.1186/s12959-021-00338-y (PMC8569956; doi:10.1186/s12959-021-00338-y)
Supplement: Supplementary file 1 — Additional file 1: Supplementary Table 1. Laboratory tests results. [file 12959_2021_338_MOESM1_ESM.docx]

Supplementary table 1. Laboratory tests results

|  | **Nov 22** | **Nov 25** | **Nov 27** | **Nov 30** | **Dec 03** | **Dec 09** | **Dec 13** | **Dec 15** | **Dec 16** | **Dec 17** | **Dec 19** | **Dec 21** | **Dec 24** |
| --- | --- | --- | --- | --- | --- | --- | --- | --- | --- | --- | --- | --- | --- |
| PT (seconds) | 10 | 12.7 | 15.9 | 12.1 | 10.8 |  | >120 | 22.2 | 15.2 | 11.6 | 9.6 | 10.1 | 10.7 |
| Fibrinogen (g /L) | 1.38 | 0.63 | 0.81 | 0.92 | 1.27 |  | <0.5 | 0.63 | 1.12 | 1.06 | 1.11 | 1.86 | 2.61 |
| FDP (mg /L) | 11.1 | 13.5 | 12.8 | 5.8 | 5.0 |  | <2.5 | 3.9 | <2.5 | <2.5 | 9.0 | 35.9 | 32 |
| D-dimer (mg /L FEU) | 6.24 | 7.48 | 7.67 | 2.51 | 2.01 |  | 0.59 | 1.75 | 0.93 | 0.78 | 4.93 | 26.93 | 17.17 |
| TAT (ng /mL) |  |  |  |  |  |  | 52 |  | 75.2 |  |  |  | 19.6 |
| PIC (μg /mL) |  |  |  |  |  |  | 0.27 |  | 0.56 |  |  |  | 2.49 |
| tPAIC (ng /mL) |  |  |  |  |  |  | 95.6 |  | 72.9 |  |  |  | 15.6 |
| Creatinine (μmol /L) | 48 | 46 |  | 44 |  |  | 55 |  | 72 | 90 | 79 |  | 31 |
| eGFR (ml /min /1.73m^2^) | 124.75 | 126.95 |  | 129.29 |  |  | 117.96 |  | 105.60 | 87.32 | 101.65 |  | 149.30 |
| Aspartate aminotransferase (IU /L) | 21 | 19 |  | 18 |  |  | 36 |  | 106 | 78 | 77 |  | 12 |
| Bilirubin (μmol /L) | 11.2 | 13.6 |  | 8.8 |  |  | 15.5 |  | 29.3 | 18.4 | 16.0 |  | 13.1 |
| PLT (× 109 /L) | 23 | 18 | 11 | 6 | 3 | 4 | 2 | 1 | 3 | 3 | 6 | 9 | 10 |
| WBC (× 109 /L) | 1.00 | 0.58 | 2.52 | 0.33 | 0.03 | 1.38 | 2.39 | 0.92 | 0.50 | 0.35 | 0.77 | 0.10 | 0.07 |
| RBC (× 1012 /L) | 2.42 | 2.35 | 2.00 | 1.99 | 2.15 | 2.18 | 2.07 | 1.75 | 1.56 | 1.57 | 2.12 | 2.54 | 2.24 |
| Hemoglobin (g /L) | 72 | 67 | 60 | 61 | 65 | 66 | 62 | 53 | 48 | 47 | 64 | 79 | 68 |
